# Importance of Preserved Tricuspid Valve Function for Effective Soft Robotic Augmentation of the Right Ventricle in Cases of Elevated Pulmonary Artery Pressure

**DOI:** 10.1007/s13239-021-00562-7

**Published:** 2021-07-14

**Authors:** Isaac Wamala, Christopher J. Payne, Mossab Y. Saeed, Daniel Bautista-Salinas, David Van Story, Thomas Thalhofer, Steven J. Staffa, Sunil J. Ghelani, Pedro J. del Nido, Conor J. Walsh, Nikolay V. Vasilyev

**Affiliations:** 1 Department of Cardiac Surgery, Harvard Medical School, Boston Children’s Hospital, 300 Longwood Ave, Boston, MA 02115 USA; 2 Clinic for Cardiovascular Surgery, Charité Universitätsmedizin, Berlin, Germany; 3 Wyss Institute for Biologically Inspired Engineering, Boston, USA; 4 Harvard School of Engineering and Applied Sciences, Boston, USA; 5 Industrial Engineering, Technical University of Cartagena, Murcia, Spain; 6 Fraunhofer EMFT, Munich, Germany; 7 Department of Anesthesiology, Critical Care and Pain Medicine, Boston Children’s Hospital, Boston, USA; 8 Department of Cardiology, Boston Children’s Hospital, Boston, MA USA

**Keywords:** Soft robotic ventricular assist device, Right heart failure, Elevated pulmonary artery pressure

## Abstract

**Purpose:**

In clinical practice, many patients with right heart failure (RHF) have elevated pulmonary artery pressures and increased afterload on the right ventricle (RV). In this study, we evaluated the feasibility of RV augmentation using a soft robotic right ventricular assist device (SRVAD), in cases of increased RV afterload.

**Methods:**

In nine Yorkshire swine of 65–80 kg, a pulmonary artery band was placed to cause RHF and maintained in place to simulate an ongoing elevated afterload on the RV. The SRVAD was actuated in synchrony with the ventricle to augment native RV output for up to one hour. Hemodynamic parameters during SRVAD actuation were compared to baseline and RHF levels.

**Results:**

Median RV cardiac index (CI) was 1.43 (IQR, 1.37–1.80) L/min/m^2^ and 1.26 (IQR 1.05–1.57) L/min/m^2^ at first and second baseline. Upon PA banding RV CI fell to a median of 0.79 (IQR 0.63–1.04) L/min/m^2^. Device actuation improved RV CI to a median of 0.87 (IQR 0.78–1.01), 0.85 (IQR 0.64–1.59) and 1.11 (IQR 0.67–1.48) L/min/m^2^ at 5 min (*p* = 0.114), 30 min (*p* = 0.013) and 60 (*p* = 0.033) minutes respectively. Statistical GEE analysis showed that lower grade of tricuspid regurgitation at time of RHF (*p* = 0.046), a lower diastolic pressure at RHF (*p* = 0.019) and lower mean arterial pressure at RHF (*p* = 0.024) were significantly associated with higher SRVAD effectiveness.

**Conclusions:**

Short-term augmentation of RV function using SRVAD is feasible even in cases of elevated RV afterload. Moderate or severe tricuspid regurgitation were associated with reduced device effectiveness.

**Supplementary Information:**

The online version contains supplementary material available at 10.1007/s13239-021-00562-7

## Introduction

In the majority of cases, right heart failure (RHF) results from a progression of left heart failure, and is associated with elevated pressures in the pulmonary circulation.^12^ Further, acute RHF may occur following LVAD Implantation.^6^ In both of the above scenarios temporary mechanical circulatory support (MCS) for the right ventricle (RV) may be considered, in cases of inadequate circulatory function despite maximal medical therapy.^7^ The goal of MCS is to reduce RV preload, while providing adequate input for the left ventricle, in order to maintain effective biventricular circulation. This may be accomplished by extra corporeal membrane oxygenation (ECMO) in the short term or implantation of a conventional right ventricular assist device (RVAD) for longer support.^2^,^5^,^9^

In recent work, our group has suggested the concept of soft robotic ventricular assist devices.^1^,^10^ Taking advantage of recent advances in soft robotics, we have shown that septally braced soft epicardial actuators can be precisely actuated in synchrony with the failing heart, to augment function.^10^ Light, compact, and with minimal blood contact, these devices provide pulsatile augmentation of the native ventricular ejection by mechanically approximating the septum and ventricular free wall, making use of the native cardiovascular chambers without the requirement to reroute blood flow.

The advantage of this approach is the considerable reduction of the surface area of foreign material that comes into contact this blood, greatly reducing the risk of thromboembolic complications. There may also be reduced cytokine stimulation and reduced shear stress on blood cells. Moreover, mimicking the native free-wall and septal approximation during systole avoids paradoxical septal motion and favors optimal hemodynamics of both the right and left ventricle.

We have previously demonstrated the feasibility of optimizing the actuation and placement of soft robotic assist devices, such that secondary mitral valve insufficiency is minimized during systole.^11^ The RV however offers a different and potentially more substantial challenge. With a relatively thin wall and bellow-like contraction, its ejection mechanics are more complicated.^4^ Longitudinal shortening and not free wall to septal approximation seems to account for the majority of right ventricular contraction.^3^ In decompensated RHF, both reduction in longitudinal contraction and ventricle chamber dilatation contribute to the derangement of ventricular hemodynamics.^4^ Further, RHF is often accompanied both by functional tricuspid regurgitation^8^ and increased pulmonary artery pressures.^13^

In this study, we describe the performance of a septally braced SRVAD in *in vivo* conditions mimicking RHF with elevated pulmonary artery pressures.

## Methods

### Setup

Nine Yorkshire swine 65–80 kg were used for the experiments. All animals received humane care according to the 1996 NIH guidelines for the care and use of laboratory animals. Boston Children Hospital’s institutional animal care and use committee reviewed and approved the study protocol.

The experiments were performed under monitored general anesthesia. The animals were fully ventilated typically in volume control mode with a positive end expiratory pressure of 4 mmHg. A lidocaine continuous infusion of 5–20 mcg/kg/minute was typically started before manipulation of the heart and maintained as prophylaxis against arrhythmia. A baseline Dopamine infusion of 2 mcg/kg/min was maintained.

Surface ECG electrodes were placed, a median sternotomy performed, and fluid filled catheters for pressure monitoring inserted into the right atrium, distal pulmonary artery and distal ascending aorta. A pressure volume catheter (Transonic AD500 PV System–Transonic Systems Inc. NY, USA) was inserted into the RV via the ventricular apex and controlled for correct placement and measurement. Ultrasonic Transonic PS flow probes (Transonic Systems Inc, NY, USA) were placed around the ascending aorta and the pulmonary artery to record the outputs of the left and right ventricles respectively. Baseline hemodynamics were then recorded using the Labchart data recording system (ADI Instruments, CO, USA). Baseline echocardiography with focus upon ventricular and valve function was performed.

### SRVAD Placement

**Figure 1:**
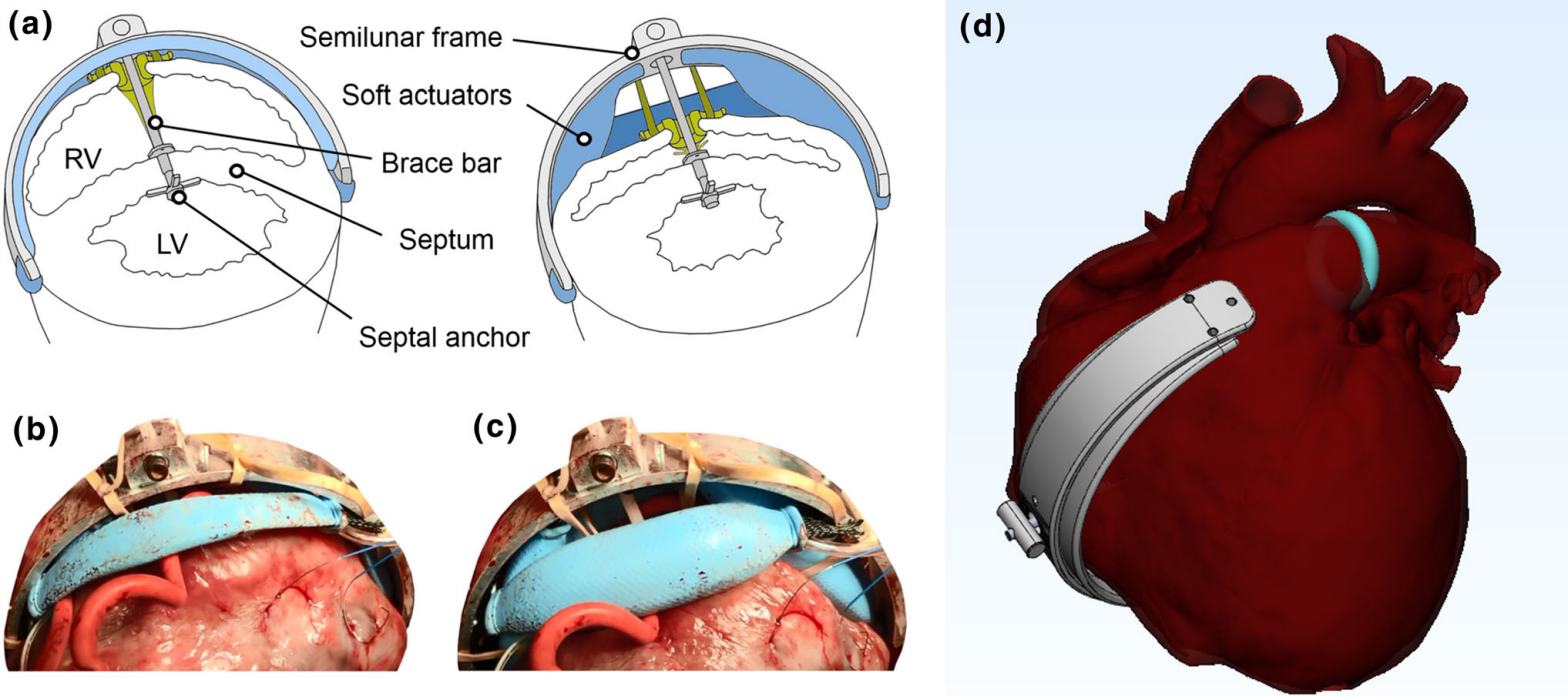
The concept of the soft-robotic right ventricular ejection device. (a). the device is made out of four Mckibben pneumatic actuators that are supported on an arced frame and braced in the interventricular septum. Contraction of the actuators approximates the septum and the free wall leading to blood ejection. (b) A prototype of the device in relaxed state. (c) the prototype in actuated state showing the contraction force applied to the RV free wall. (Adopted with permission from Payne *at al.* Science Robotics, 22 Nov 2017). (d) An illustration of the right heart failure model. We applied a pulmonary artery (PA) band to cause RHF leaving the band in place during actuation.

**Figure 2:**
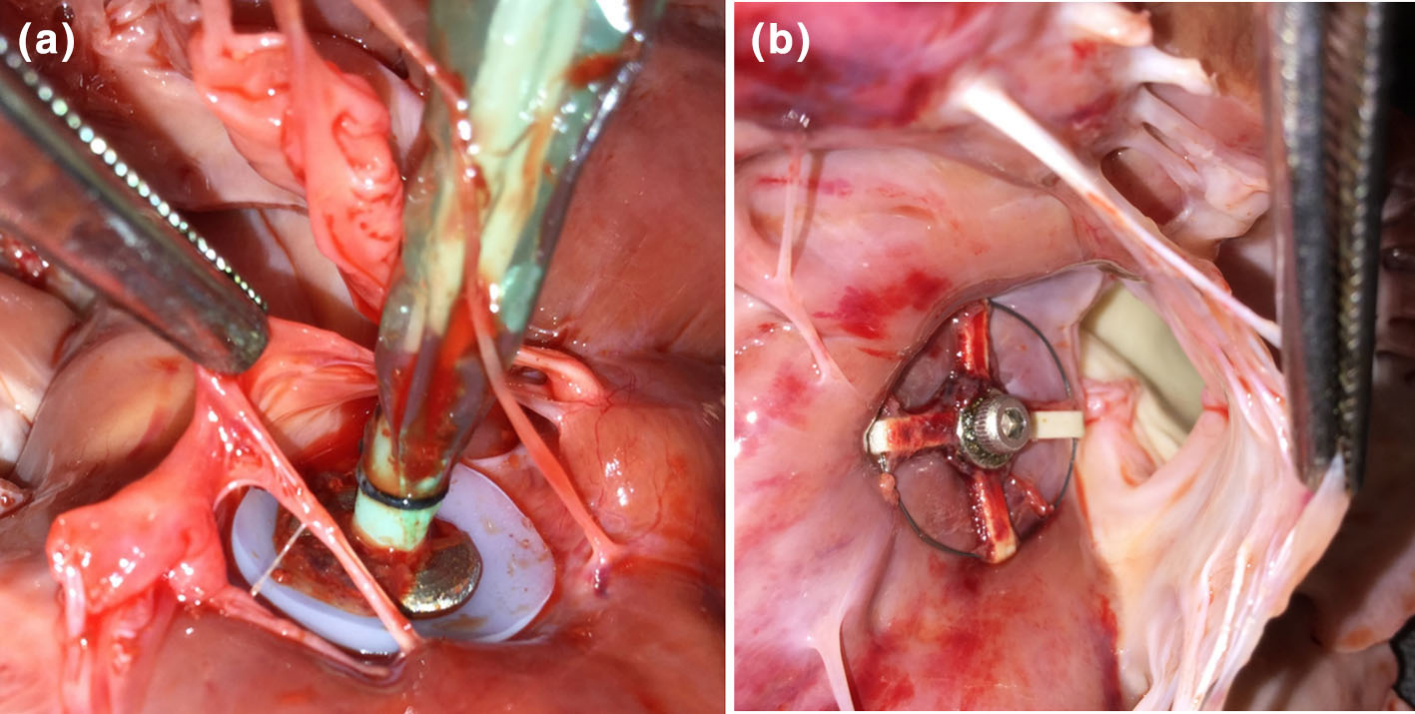
Examination of the explanted heart. (a) View from the right side of the intraventricular septum. (b) The view from the left side of the intraventricular septum. There was no apparent damage to the septum or intracardiac structures after an hour of device actuation.

The SRVAD was placed under echocardiographic guidance on the beating heart, as previously described.^10^ Briefly, the device is composed of a septal anchoring system, an arc profile mimicking the right ventricular curvature, on which soft robotic actuators are mounted, a semi-flexible rod linking the arc to the anchoring system, and a blood isolation system to keep the device from direct contact with blood and prevent leaking of blood from the RV (Fig. 1). SRVAD is implanted on the beating heart under 3D echocardiography guidance. An area of RV free wall at the level of the papillary muscle is chosen and a purse-string is placed. Within the purse-string suture, an introducer needle is inserted through the RV and intraventricular septum into the left ventricle. The ideal area of intraventricular septum is the smooth area just inferior to the septal band and just apical to the outflow septum. This insertion point is well away from both the right and left electrical conduction bundles and is also ideal for engagement of the septum by the SRVAD. On the left side the needle would typically end up adjacent to the left ventricular outflow tract and avoid the mitral valve chordae (Fig. 2). Once the needle position is confirmed, the Seldinger technique is used to introduce the septal anchoring system which is then unfolded for firmly sandwich both sides of the IVS (Figs. 1 and  2). The RV seal system is then applied, and the anchor connected to the actuator arc via the semi-flexible rod. A second baseline of hemodynamic and echocardiographic data were recorded prior to creation of RHF.

### Right Heart Failure Model

Acute RHF was created by pulmonary artery banding. A cotton umbilical tape 0.32 cm wide (Ethicon-Johnson and Johnson, NJ, USA) was used for umbilical banding. For the purposes of the experiment, the tape was marked into 2 mm. lengths to allow precise reduction of the pulmonary artery circumference. The band was placed around the proximal main pulmonary artery at least 2 cm distal to the pulmonary valve and proximal to the flow and pressure probes. The position was maintained by fixing the band to the posterior pulmonary artery wall using a 5/0 prolene stitch. Using a tourniquet system, the main pulmonary artery lumen is gradually occluded by tightening the band in 2-mm increments. RHF is achieved once the cardiac output falls below 60% of baseline. The band is left in place and after allowing a period of stabilization, the RHF baseline data is recorded.

### SRVAD Actuation

SRVAD was then actuated in synchrony with the heart rhythm based on detection of the RV pressure signal. The supplementary video material shows a video of the RVAD actuation. SRVAD triggers just as the RV pressure begins to build at the beginning of systole and remains actuated for 35% of the cardiac cycle. SRVAD actuators relax in synchrony with the heart. Hemodynamics were recorded at 5, 30 and 60 min of actuation.

### Echocardiography

All echocardiograms were reviewed and scored by a single observer experienced in cardiac imaging. Tricuspid regurgitation grade was categorized as 0 = None or Trivial, 1 = Mild, 2 = Moderate and 3 = Severe TR. In-between grades were denoted by a 0.5 added to the lower grade. Video 2 demonstrates an example of an animal with trivial tricuspid regurgitation and another with severe tricuspid regurgitation following RHF creation.

### Statistical Analysis

Data from the periods; baseline, device implanted, right heart failure, 5, 30 and 60 min actuation were collected. In each period, representative data covering at least 15 s duration was extracted from the continuous hemodynamic traces, using the Labchart program. Because of the variation in animal size, cardiac output and stroke volume were adjusted to BSA to generate a cardiac index (CI) and stroke volume index (SVI). Continuous variables were summarized as median and interquartile range and categorical variables as frequencies and percentages. Comparisons of continuous data between specific time points were done using the Wilcoxon signed ranks test. Generalized estimating equation (GEE) was used to model the change in each parameter over time while taking into account repeated measurements within an animal and adjusting for BSA, while implementing a Gaussian family and identity link function for continuous outcome data. All statistical analyses were performed using Stata version 15.0 (StataCorp LLC., College Station, Texas). A two-tailed significance threshold of *p* < 0.05 was used to determine statistical significance.

## Results

### Right Ventricle Augmentation

**Figure 3:**
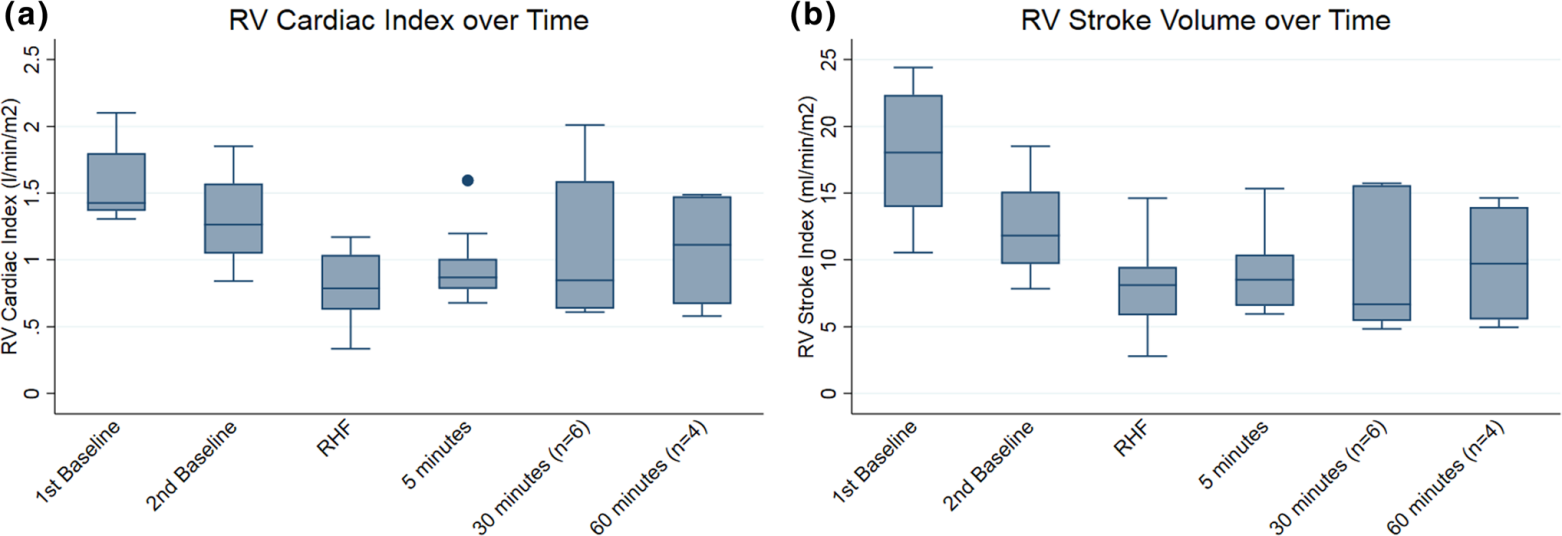
The overall effects of the device on RV function. (a) RV Cardiac Index at the different time points of the experiment. There was a significant increase in cardiac index at 30 min and 60 min of actuation compared to the RHF baseline. (b) RV Stroke Volume Index at the different time points during the experiment.

One-hour RV support was completed in four animals. In five animals 5–30 min support was completed. The effect of SRVAD actuation on RV cardiac index and RV stroke volume index are illustrated in Fig. 3. Median RV cardiac index was 1.43 (IQR, 1.37–1.80) L/min/m^2^ at the first baseline and 1.26 (IQR 1.05–1.57) L/min/m^2^ at second baseline measurement. Upon PA banding RV cardiac index fell to a median of 0.79 (IQR 0.63–1.04) L/min/m^2^. SRVAD actuation resulted in an improvement of RV cardiac index to a median of 0.87 (IQR 0.78–1.01), 0.85 (IQR 0.64–1.59) and 1.11 (IQR 0.67–1.48) L/min/m2 at 5 min (*p* = 0.114), 30 min (*p* = 0.013) and 60 min (*p* = 0.014) respectively. The median RV stroke volume index at first and second baseline measurement were 18 (IQR 14–22) mL/beat/m^2^ and 12 (IQR 10–15) mL/beat/m^2^ respectively. RV stroke volume at the point of RHF was 8 (IQR 6–10) mL/beat/m^2^. After device actuation, median stroke volume index was 9 (IQR 7–10) mL/beat/m^2^ at 5 min (*p* = 0.198); 7 (IQR 5–16) mL/beat/m^2^ at 30 min (*p* = 0.273); and 10 (IQR 6–14) mL/beat/m^2^ at 60 min (*p* = 0.286) of actuation. There was a positive correlation between the RV cardiac index improvement at 5 min to subsequent timepoints at 30 min (r = 0.314, *p* = 0.544) and one hour (r = 0.8, *p* = 0.2). Similarly, augmentation in RV stroke volume at 5 min was associated with the subsequent augmentation at 30 min (*r* = 0.486 *p* = 0.329) and 1 h (*r* = 0.8, *p* = 0.2).

### Left Ventricle Augmentation

**Figure 4:**
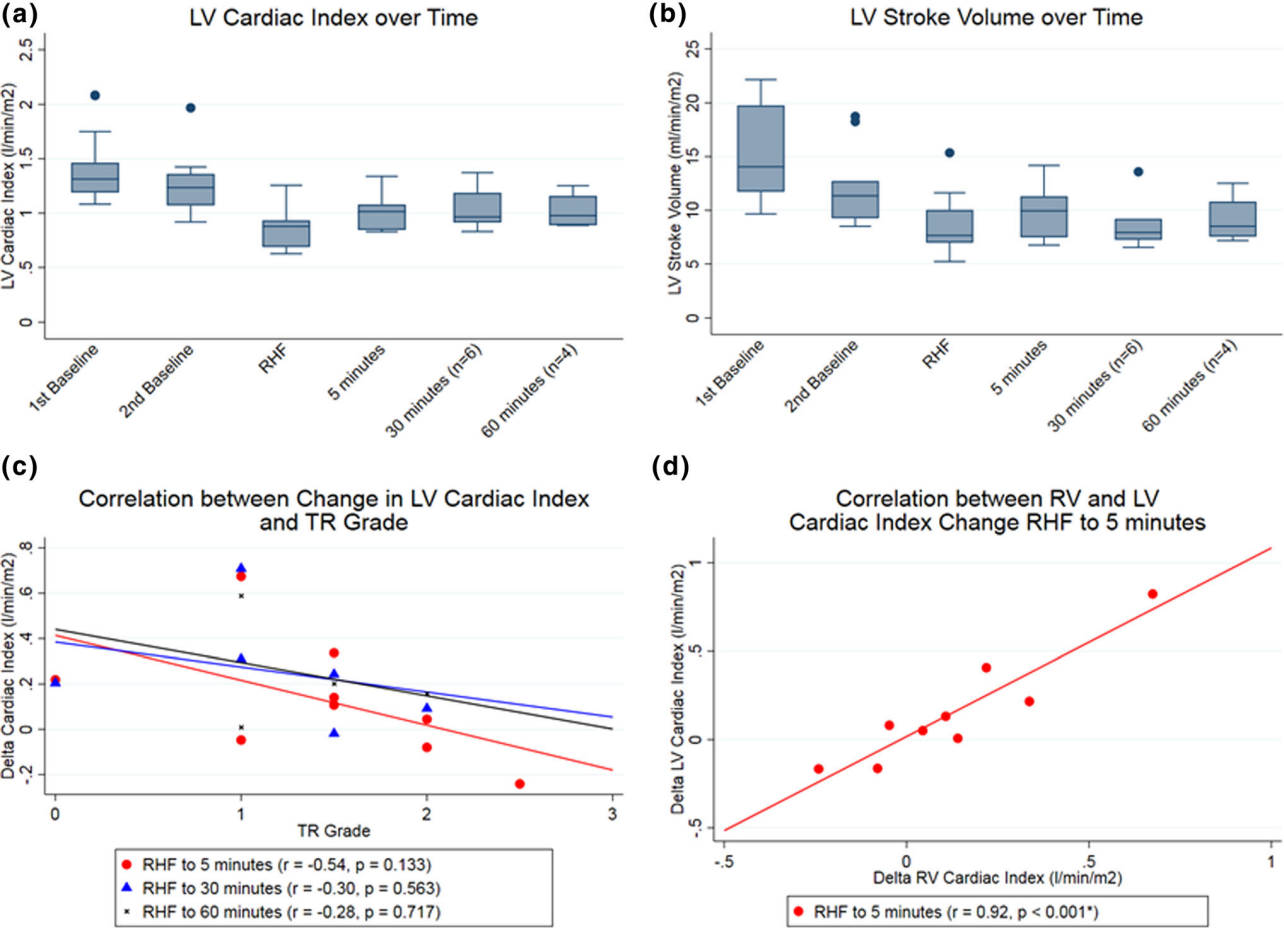
Effect of the device on the left ventricle. (a) LV cardiac index at the different experimental time-points. (b) LV stroke volume index at the different time-points during the experiment. (c) Correlation between change in LV cardiac index and grade of tricuspid regurgitation (d) Correlation between right ventricle and left ventricle cardiac index change at 5 min of device actuation.

The effect of the device on left ventricle function is summarized in Fig. 4. The median LV cardiac index was 1.31 (IQR 1.19–1.46) L/min/m^2^ first baseline and was 1.23 (IQR 1.07–1.36) L/min/m^2 at^ second baseline. Median LV CI fell to 0.88 (IQR 0.69–0.92) L/min/m^2^ upon creation of RHF. LV cardiac index at was 1.01 (IQR 0.85–1.08), 0.96 (IQR 0.91–1.19) and 0.98 (IQR 0.89–0.98) L/min/m^2^ at 5 min (*p* = 0.132), 30 min (*p* = 0.019) and 60 min (*p* = 0.033) of device actuation respectively. LV stroke volume index was 14 (IQR 12–20) mL/beat m^2^ at first baseline and was 11 (IQR 9–13) mL/beat/m^2^ at second baseline measurement. Stroke volume index reduced to 8 (IQR 6–9) mL/beat/m^2^ upon RHF creation and was 10 (IQR 7–11) mL/beat/m^2^, 8 (IQR 7–9) mL/beat/m^2^ and 9 (IQR 8–11) mL/beat/m^2^ at 5, 30 and 60 min of device actuation. Overall LV effective LV augmentation was achieved in animals where effective RV augmentation was also achieved (Fig. 4).

### Pulmonary Artery Pressures

Mean PA pressure was 15 (IQR 14–16) mmHg at first baseline, 21 (IQR 19–24) mmHg at second baseline measurement. Mean PA pressure, measured distal to the PA band, was 18 (IQR 14–22) mm Hg at RHF and remained relatively unchanged during the device actuation at 20 (IQR 16–21) mmHg, 21 (IQR 18–25) mmHg and 19 (IQR 18–21) mmHg at 5, 30 and 60 min respectively. Maximal PA pressure increased from a 23 (IQR 21–27) mmHg at RHF to 27 (IQR 23–32) mmHg, 30 (IQR 27–32) mmHg and 28 (IQR 27–29) mmHg, 5, 30 and 60 min of device actuation. The change in maximal PA pressure was statistically significantly different from the RHF reading within 5 min of device actuation (*p* = 0.027) but was not significant at 30 min (*p* = 0.09) and 60 min (*p* = 0.875).

### Right Ventricle Pressures

RV end diastolic pressures were 4 (IQR 3–5) mmHg, 6 (IQR 6–8) mmHg, 7 (IQR 3–8) mmHg, 6 (IQR 2–8) mmHg, 5 (IQR 4–7) mmHg and 3 (IQR 2–6) mmHg at first baseline, second baseline, RHF, 5, 30 and 60 min actuation respectively. Maximal RV pressure was 22 (IQR 19–27) mmHg at baseline and 28 (IQR 27–33) mmHg firsts and second baseline measurement respectively. Upon RHF creation maximal RV pressure was 32 (27–35) mmHg. Maximal RV pressure increased to 38 (IQR 35–43) mmHg at 5 min (*p* = 0.012), 36 (IQR 32–43) mmHg at 30 min (*p* = 0.063) and 34 (IQR 30–36) mmHg at 60 min actuation (*p* = 0.875).

### Systolic Pressure

Median systolic pressure was 82 (IQR 74–89) and 56 (IQR 51–62) mmHg at first and second baseline measurement, and it was 43 (IQR 39–49) mmHg at RHF. Systolic pressure was 49 (IQR 42–55) mmHg, 47 (IQR 46–49) mmHg and 47 (IQR 42–53) mmHg at 5 min (*p* = 0.098), 30 min (*p* = 0.094) and 60 (*p* = 375) min of device actuation.

### Mean Arterial Pressures

Mean arterial pressure was 62 (IQR 57–62) mmHg at first baseline and 46 (IQR 35–49) mmHg at second baseline. The mean arterial pressure fell to 34 (IQR 29–37) mmHg upon RHF creation and was 37 (IQR 34–38) mmHg at 5 min (*p* = 0.496), 34 (IQR 33–36) mmHg at 30 min (*p* = 0.438) and 34 (IQR 32–38) mmHg at 60 min actuation (*p* = 0.375).

### RV Pressure/Systemic Pressure

The fraction of RV/Systemic fraction was 0.26 (0.25–0.29) and 0.57(0.49–0.64) on first and second baseline measurements and was 0.73 (0.7–0.77) at RHF. The RV/Systemic fraction was 0.78 (0.76–0.82), 0.78 (0.68–0.91) and 0.65 (0.59–0.81) at 5 min (*p* = 0.004), 30 min (*p* = 0.688) and 60 min respectively (*p* = 0.875).

### Tricuspid Regurgitation

At the time of RHF prior to device actuation, one animal had trivial tricuspid regurgitation, two animals had mild tricuspid regurgitation, 3 animals mild + tricuspid regurgitation and the remaining three animals had moderate or worse tricuspid regurgitation.

**Figure 5:**
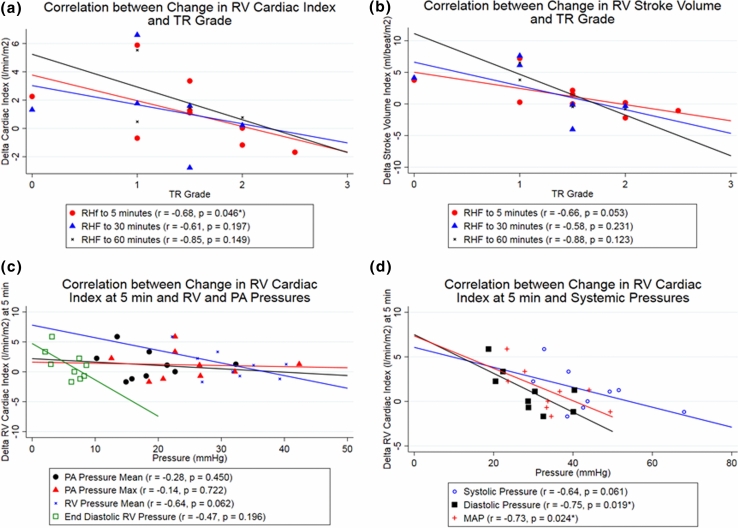
Correlation between changes in cardiac index and strove volume index to tricuspid function, RV and PA pressures. (a) The correlation of RV cardiac index changes at 5, 30 and 60 min of device actuation with the grade of tricuspid regurgitation. (b) Correlation between change in RV stroke volume index and tricuspid regurgitation grade (c) Correlation between change in RV cardiac index at 5 min and RV and PA pressures. (d) Correlation between change in RV cardiac index at 5 min and systemic pressures.

The relationship of RV cardiac output change to tricuspid regurgitation is shown in Fig. 5. Improved RV cardiac index at 5 min (*p* = 0.046), 30 min (*p* = 0.197), and 60 min (*p* = 0.149) of device actuation is associated with a lower grade of tricuspid regurgitation (TR). Similarly stroke volume improvement at 5 min (*p* = 0. 053), 30 min (*p* = 0.231), and 60 min (*p* = 0.123) of device actuation tended to be better at low grades of TR. A lower systemic diastolic pressure (*p* = 0.019) and lower mean arterial pressure (*p* = 0.024) were associated with improved RV cardiac index change (Fig. 5d).

## Discussion

For patients with persistent symptomatic RHF despite maximal medical therapy, the main MCS options in current practice are ECMO implantation for short-term support, or a conventional RVAD Implantation, often in BiVAD configuration with an LVAD, for longer-term support. The concept of a SRVAD, with septally braced cardiosychronic epicardial actuators, has been suggested as an alternative therapy.^10^ By making use of the native cardiovascular circuit and mimicking native ventricular mechanics, these SRVADs have a minimal surface area of foreign material in contact with blood. They act primary by increasing the contractile force during systole and augmenting diastolic relaxation.

SRVADs necessitate two important shifts in the clinical approach to VAD therapy. Unlike the current MCS devices, SRVADs were conceived to augment and not completely replace function of a failing ventricle. Secondly, making use of the native cardiovascular circuit, SRVADs require competence of the native one-way cardiac valves for efficient forward ejection.

In our prior work, we have demonstrated that a SRVAD not only augments left ventricular function but also concurrently acts on the mitral valve to reduce mitral valve insufficiency during actuation.^11^ In the current experiments, the device itself did not increase or reduce the tricuspid valve function. We observed augmentation of RV output to the levels of the second baseline prior to RHF or better, only in some animals, despite evidence that the device was able to augment RV contraction, as evidenced by the increase in RV and maximal PA pressures. Efficient forward ejection in the presence of a pulmonary band was associated with the level of tricuspid valve competence at the time of RHF. Further, we observed a difference in device function at the 30 min and one-hour timepoints among those animals with good initial improvement in cardiac function which tended to maintain this improvement compared to those without good initial improvement whereby the cardiac output further worsened with time, because of ongoing RHF without effective device augmentation.

## Study Limitations

Acute heart failure creation by pulmonary banding in one stage does not precisely recreate the hypertrophy seen in RHF following chronically elevated pulmonary pressures. Further, the mean arterial pressure and diastolic pressure may have an influence on RV filling although we did not directly measure this factor.

## Conclusions

This work is an early translational undertaking that builds on our initial proof of concept studies, to evaluate the concept of a soft robotic right ventricular assist device in a setting of right heart failure and elevated pulmonary artery pressures. We demonstrate that hemodynamically relevant right ventricular support for up to an hour is feasible. We find that significant tricuspid regurgitation hinders effective forward ejection. The next translational step would be studies of 6 to 72 h support using these devices and in animal models where right heart failure is induced over weeks and not over minutes, in order to more closely simulate the intended clinical use and to study the physiological changes in detail. The data from this study suggest that addressing tricuspid valve function, either in the design of the devices or as a concomitant procedure, will be a necessary step prior to further clinical translation.

## Supplementary Information

Below is the link to the electronic supplementary material.

Video 1: A video showing the SRVAD device implanted on the heart. An epicardial and echocardiographic view of the failing right heart following pulmonary band placement, with and without device actuation. Supplementary material 1 (MP4 9353 kb)

Video 2: A video showing the SRVAD actuation in an animal with trivial tricuspid regurgitation following right heart failure creation and anther with severe tricuspid regurgitation. Supplementary material 2 (MP4 2763 kb)

## Data Availability

Data from the experiments described in this study is available for sharing with other investigators.

## Abbreviations

CI: Cardiac index
PA: Pulmonary artery
RVAD: Right ventricular assist device
RHF: Right heart failure
SRVAD: Soft robotic right ventricular assist device
SVI: Stroke volume index

## References

[1] Bautista-Salinas D (2020). Synchronization of a soft robotic ventricular assist device to the native cardiac rhythm using an epicardial electrogram. J. Med. Device..

[2] Bernhardt AM, De By TMMH, Reichenspurner H, Deuse T (2015). Isolated permanent right ventricular assist device implantation with the heartware continuous-flow ventricular assist device: first results from the european registry for patients with mechanical circulatory support. Eur. J. Cardio-thoracic Surg..

[3] Brown SB, Raina A, Katz D, Szerlip M, Wiegers SE, Forfia PR (2011). Longitudinal shortening accounts for the majority of right ventricular contraction and improves after pulmonary vasodilator therapy in normal subjects and patients with pulmonary arterial hypertension. Chest.

[4] Buckberg G, Hoffman JIE (2014). Right ventricular architecture responsible for mechanical performance: unifying role of ventricular septum. J. Thorac. Cardiovasc. Surg..

[5] Cheung AW, White CW, Davis MK, Freed DH (2014). Short-term mechanical circulatory support for recovery from acute right ventricular failure: clinical outcomes. J. Heart Lung Transpl..

[6] Hayek S, Sims DB, Markham DW, Butler J, Kalogeropoulos AP (2014). Assessment of right ventricular function in left ventricular assist device candidates. Circ. Cardiovasc. Imaging.

[7] Kapur NK (2017). Mechanical circulatory support devices for acute right ventricular failure. Circulation..

[8] Lee CH (2019). Mechanics of the tricuspid valve—from clinical diagnosis/treatment, in vivo and in vitro investigations, to patient-specific biomechanical modeling. Bioengineering..

[9] Noly PE (2014). Temporary right ventricular support following left ventricle assist device implantation: a comparison of two techniques. Interact. Cardiovasc. Thorac. Surg..

[10] Payne CJ (2017). Soft robotic ventricular assist device with septal bracing for therapy of heart failure. Sci. Robot..

[11] Saeed MY (2020). Dynamic augmentation of left ventricle and mitral valve function with an implantable soft robotic device. JACC Basic Transl. Sci..

[12] Voelkel NF (2006). Right ventricular function and failure: report of a National Heart, Lung, and Blood Institute working group on cellular and molecular mechanisms of right heart failure. Circulation.

[13] Vonk Noordegraaf A (2019). Pathophysiology of the right ventricle and of the pulmonary circulation in pulmonary hypertension: an update. Eur. Respir. J..

